# Ultrasound of the Uterosacral Ligament, Parametrium, and Paracervix: Disagreement in Terminology between Imaging Anatomy and Modern Gynecologic Surgery

**DOI:** 10.3390/jcm10030437

**Published:** 2021-01-23

**Authors:** Marco Scioscia, Arnaldo Scardapane, Bruna A. Virgilio, Marco Libera, Filomenamila Lorusso, Marco Noventa

**Affiliations:** 1 Unit of Gynecological Surgery, Mater Dei Hospital, 70125 Bari, Italy; 2 Section of Diagnostic Imaging, Interdisciplinary Department of Medicine, University of Bari “Aldo Moro”, 70100 Bari, Italy; arnaldo.scardapane@gmail.com (A.S.); milalorusso@yahoo.it (F.L.); 3 Department of Obstetrics and Gynecology, Policlinico Hospital, 35031 Abano Terme, Italy; bruna81@tiscali.it (B.A.V.); marcolibera@yahoo.it (M.L.); 4 Department of Women and Children’s Health, Clinic of Gynecology and Obstetrics, University of Padua, 35121 Padua, Italy; marco.noventa@gmail.com

**Keywords:** endometriosis, cervical cancer, ultrasound, anatomy, ureter, nerves, hypogastric nerve

## Abstract

Ultrasound is an effective tool to detect and characterize lesions of the uterosacral ligament, parametrium, and paracervix. They may be the site of diseases such as endometriosis and the later stages of cervical cancer. Endometriosis and advanced stages of cervical cancer may infiltrate the parametrium and may also involve the ureter, resulting in a more complex surgery. New functional, surgical anatomy requires the complete diagnostic description of retroperitoneal spaces and tissues that contain vessels and nerves. Most endometriosis lesions and cervical cancer spread involve the cervical section of the uterosacral ligament, which is close to tissues, namely the parametrium and paracervix, which contain vessels and important nerves and nerve anastomoses of the inferior hypogastric plexus. Efferent fibers of the plexus travel to the rectum, uterus, rectovaginal ligament, deep vesicouterine ligament, and bladder. These efferent fibers are essential for bladder and rectal functionality so tailored nerve-sparing surgery became a standard approach for treating deep infiltrating endometriosis and cervical cancer. An accurate diagnosis by ultrasound has significant clinical impact and is important for appropriate treatment. In this article, we try to establish a common terminology between imaging diagnostic and modern surgical anatomy.

## 1. Introduction

The uterosacral ligaments (USLs) are extraperitoneal structures that extend backward from the posterior surface of the cervix and upper vagina to the second-to-fourth sacral vertebrae, forming the lateral boundaries of the rectouterine and rectovaginal spaces. They are composed mainly of connective tissue along with vessels and splanchnic nerve fibers [1]. The USLs are surrounded by the paracervical and parametrial tissues, which are fibrous and fatty connective tissues rich with vessels and nerves. These structures can be clinically evaluated by a combined rectovaginal examination and by ultrasound, and they may be the site of diseases such as endometriosis and the later stages of cervical cancer.

Endometriosis is a common, chronic, and debilitating gynecological condition that affects between 5% and 15% of women of reproductive age [2,3]. It is characterized by the growth of tissue that looks and acts like endometrial tissue, exhibiting the same response to hormonal changes; however, this abnormal tissue growth occurs outside the uterus, usually on other organs inside the pelvis and in the abdominal cavity. These growths, called endometriosis implants, lead to local inflammatory reactions that, in turn, promote a fibrotic reaction and the formation of adhesions, which prevent organs from sliding along each other normally and can result in an altered normal pelvic anatomy [4,5,6]. This cascade of events often causes menstrual and/or chronic pelvic pain, infertility, or malfunction of the affected abdominopelvic organs [2].

Endometriosis of the uterosacral ligament (USL) is a leading cause of deep dyspareunia and pelvic pain [7,8], and large endometrial nodules may also involve the parametrium and the ureter, resulting in a more complex surgery [4,9]. The posterior pelvic compartment, and the USL specifically, have been reported to be among the most common locations for endometriosis lesions [10,11]. In a study of patients with all stages of endometriosis, Chapron et al. [11] reported a prevalence of USL endometriosis of about 69%. In 83% of cases, USL endometriosis was found as an isolated lesion (study population 241, number of lesions 344) [11]. We previously studied a large cohort of patients with ASRM-stage IV endometriosis (study population 1548; number of lesions 10,466) [10,12] and reported a USL endometriosis prevalence of about 52%, with a parametrial/paracervical involvement in 25% of cases.

Cervical cancer arises from the cervix and may invade adjacent structures, such as the vagina and the paracervical tissue, as it progresses. From the International Federation of Gynecology and Obstetrics (FIGO) stage IIB onwards, the cancer may spread further to the USLs and to more distant organs [13]. The estimated incidence of cervical cancer is of 15.7 per 100,000 women, although incidence varies widely among countries, with a generally high prevalence of early-stage cervical cancer due to cervical screening programs [14]. Extracervical cancerous lesions require a more aggressive treatment and are associated with higher case-fatality rates [14,15].

Here, we discuss the potential for using ultrasound to detect endometrial or cancerous lesions, and we emphasize the importance of a correct diagnosis for optimizing treatment (modern functional surgery) and preventing severe surgical complications.

## 2. Anatomical Terminology

Discrepancies between the anatomical terminology used in imaging diagnostic and modern gynecological surgery was reported [16,17]. Surgeons have developed a different nomenclature from classical anatomical terminology for pelvic retroperitoneal spaces and the connective tissue bundles to describe in detail surgical procedures [16,17,18]. In ultrasound and magnetic resonance imaging (MRI), the pelvic connective tissue covered with the peritoneum is identified in general as parametrium and cardinal and uterosacral ligaments and this hinders communication between diagnosis and treatment of pelvic pathologies. In fact, anatomical structures that are of potential clinical interest for functional-sparing surgery (i.e., the paracervical tissue) are often neglected in imaging reports.

The purpose of this paragraph was to meet pelvic diagnostic images with new surgical anatomy.

### 2.1. The Uterosacral Ligament

**Figure 1:**
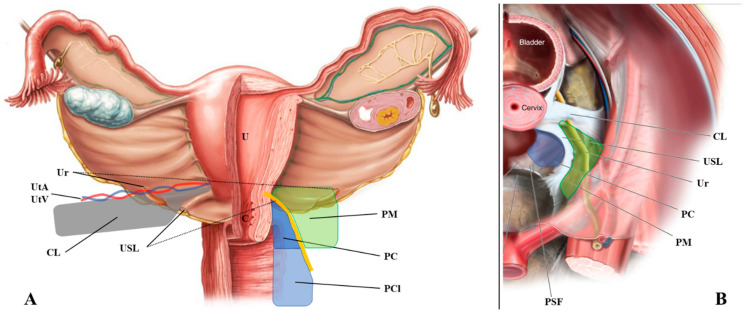
Anatomical definition of the uterosacral ligament, parametrium, paracervix, and paracolpium. (**A**) shows the frontal and (**B**) the upper view of the pelvic posterior compartment. Abbreviations: C, cervix; CL, cardinal ligament; PC, paracervix; PCl, paracolpium; PM, parametrium; PSF, presacral fascia; R, rectum; U, uterus; Ur, ureter; USL, uterosacral ligament; UtA, uterine artery; UtV, uterine vein.

The uterosacral ligaments are retroperitoneal structures that extend posteriorly from the uterine cervix to sacrum, defining the lateral boundaries of the rectouterine and rectovaginal spaces (Figure 1). From an anatomical perspective, the USL can be divided into three parts: cervical, intermediate, and sacral sections [1]. The cervical section is composed of dense connective tissue containing small blood vessels and small branches of the inferior hypogastric plexus (IHP).

### 2.2. The Parametrium

The parametrium is the fibrous and fatty connective tissue that surrounds the uterus. It is bordered laterally by the internal iliac vessels, medially by the uterus, superiorly by the peritoneum, and inferiorly by the ureter. This tissue contains the uterine artery and the superficial uterine vein [17].

### 2.3. The Paracervix

The paracervix is the fibrous and fatty tissue that lies beneath the parametrium (Figure 1) [18]. Like the parametrium, it is bordered laterally by the internal iliac vessels, but its medial border is the upper two-thirds of the vaginal wall and the insertion of the USL. It is the upper part of the paracolpium that contains important functional nerves and vessels. The upper limit of the paracervix is the ureter and the lower limit is the levator ani muscle and the presacral fascia. The paracervix contains the inferior vesical artery, the vaginal artery, the proximal part of the deep uterine vein, the pelvic splanchnic nerves, the distal part of the hypogastric nerve, and the IHP.

## 3. What Ultrasound Should Investigate and Detect

Ultrasound visualization of the USL can be obtained using a transvaginal approach [19]. These images can be used to assess the presence of disease and determine if there is any parametrial involvement. For this procedure, the ultrasound probe is placed in the posterior vaginal fornix in longitudinal axis and then rotated laterally of about 45°, showing the hyperechoic USL. Disease (either endometriosis or cancer) appears as a hypoechoic lesion that alters the regular (fibrotic) pattern of the ligament. Parametrial involvement may be seen as an extension (same echogenicity) of the primary lesion more distant from the probe.

**Figure 2:**
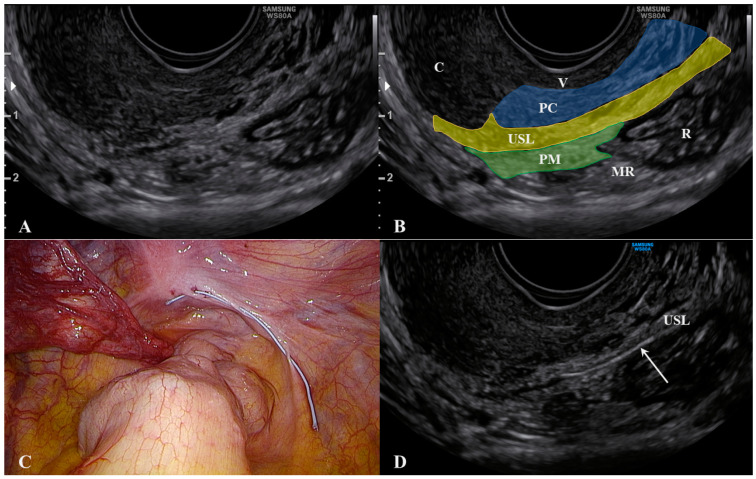
Ultrasound visualization of the uterosacral ligament, paracervix, and parametrium. Figure (**A**) shows the normal appearance and explanations are reported in figure (**B**). A polyester suture was placed on a physiological USL during laparoscopy (figure (**C**) and identified via ultrasound (arrow) in figure (**D**). Abbreviations: C, cervix; MR, mesorectum; PC, paracervix; PM, parametrium; R, rectum; USL, uterosacral ligament; V, vagina wall.

The cervix should appear on one side of the image to ensure that the edge of the ligament that is closest to the cervix can be assessed. The first important consideration during ultrasound of the USL is to not push the probe upwards while it is correctly placed. This method allows for clear visualization of not only the USL, but also the parametrium (distal to the probe) and, most importantly, the paracervix (proximal to the probe). As shown in Figure 2, we placed a 5 Ti-CronTM polyester suture (a heavy braided suture for orthopedics, diameter 0.7 mm) along a physiological USL during surgery to confirm the ultrasound visualization of the ligament. In this way, we could simultaneously visualize the vaginal wall, the paracervical tissue, the USL, and the parametrium. All structures could be visualized from the probe to more distant regions (Supplementary Materials Video S1).

**Figure 3:**
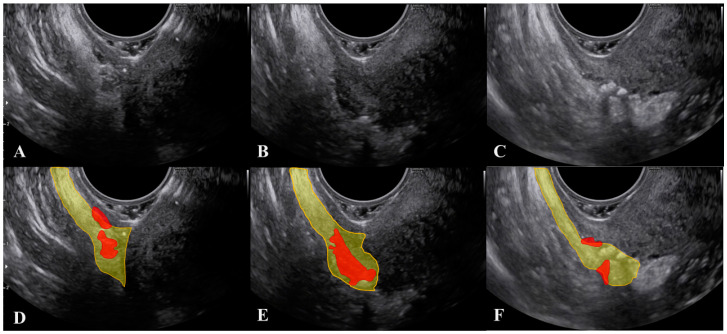
Modification of the visualization of lesions of the uterosacral ligament during the ultrasound when the probe is pushed. Figure 3 (**D**–**F**) represent the marked images respectively of figure (**A**–**C**). The uterosacral ligament is marked in yellow and lesions in red.

Another important consideration is that, during the ultrasound, strongly pushing the probe to make the USL closer to the vagina may result in a displacement (rotation) of the ligament, which can mask the actual extent of the lesion (Figure 3 and Supplementary materials Video S2).

**Figure 4:**
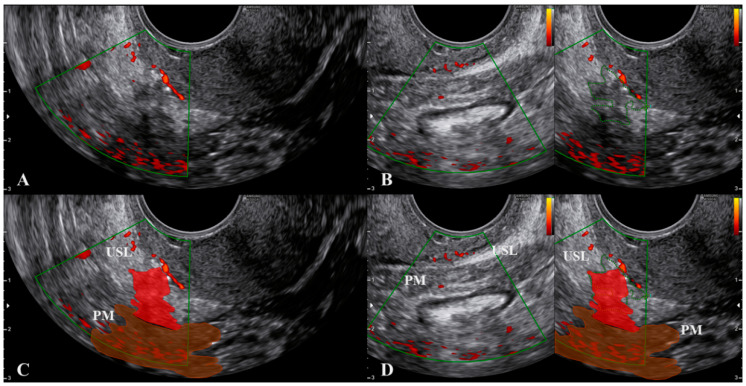
Visualization of the small vessels within the lowest part of the uterosacral ligament using color Doppler. Figure (**C**,**D**) are the marked images of figure (**A**,**B**), respectively. Endometriosis lesions of the uterosacral ligament and parametrium are in red. Abbreviations: PM, parametrium; USL, uterosacral ligament.

**Figure 5:**
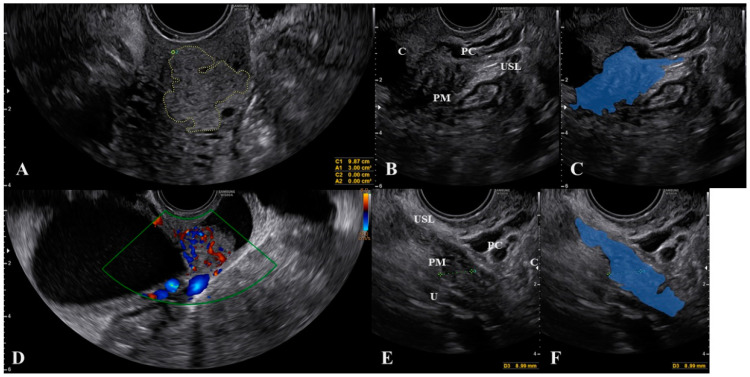
Cervical carcinoma (**A**,**D**) that spreads to the uterosacral ligaments (**B**) as marked in figure (**C**). Figure (**E**) demonstrate the uterosacral spread of the cancer as marked in figure (**F**). Abbreviations: C, cervix; PC, paracervix; PM, parametrium; U, ureter; USL, uterosacral ligament.

To obtain a correct visualization of both the USL and the paracervical and parametrial tissues, we suggest not only relying on the subjective feeling of the operator (i.e., not pushing), but also visualizing the small vessels within the lowest part of the USL using color Doppler (Figure 4 and Supplementary Materials Video S3). These thin vessels may be temporarily closed by pushing the probe. Ligament rotation and vessel disappearance are particularly evident for endometriosis lesions but are less pronounced in cases of cervical cancer spread, as the latter lesions are integral with the cervix (Figure 5) and the cervix is less easy to rotate.

**Figure 6:**
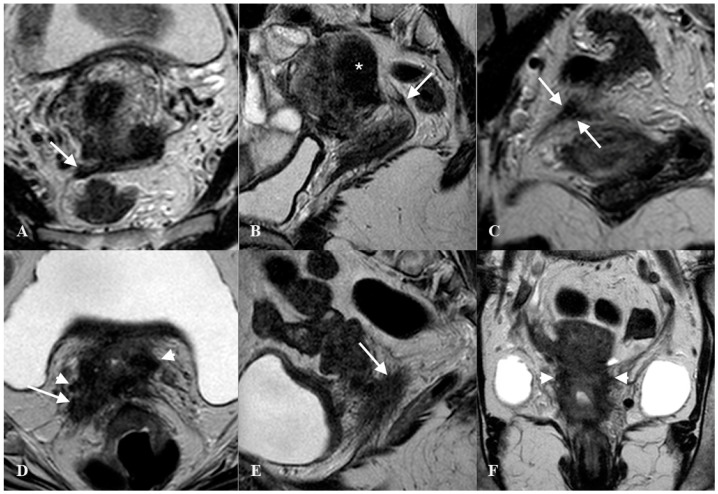
Thickened right uterosacral ligament in a woman with deep infiltrating endometriosis and adenomyosis. Axial HR T2w-Turbo Spin Echo (TSE) image (**A**), Sagittal HR T2w-TSE image (**B**), Coronal HR T2w-TSE image (**C**). The right uterosacral ligament shows a nodular thickening (arrows). Diffuse adenomyosis of posterior uterine wall (*). Advanced cervical cancer infiltrating right uterosacral ligament (arrow) and parametrium (arrowhead). Axial HR T2w-Turbo Spin Echo (TSE) image (**D**), Sagittal HR T2w-TSE image (**E**), Coronal HR T2w-TSE image (**F**).

The diagnostic accuracy for endometriosis lesions evaluated using ultrasound has been reported as moderate (sensitivity 0.67, specificity 0.86) [20], which is similar to the accuracy of MRI (sensitivity 0.70, specificity 0.93) [21]. Alcazar et al. [22] assessed parametrial infiltration in cervical cancer and reported a similar diagnostic accuracy for ultrasound (sensitivity 0.78, specificity 0.96), which was also comparable to the performance of MRI. For many years, MRI was the diagnostic gold standard for assessing the extent of lesions palpated with a bimanual combined rectovaginal examination (Figure 6).

Recently, fusion imaging has gained interest for determining USL and parametrial involvement in endometriosis and cervical cancer spread [23,24,25]. Fusion imaging, also referred to as real-time virtual sonography, displays live ultrasound imagery and previously-acquired MRI images side-by-side on a single screen. This technique requires an average of 13 min to perform [24], but early work has shown that it may improve the detection rate of parametrial involvement [23,24]. Larger studies are required to confirm these pivotal findings. One of the main advantages of fusion imaging is the visualization of peripheral nerves (i.e., the hypogastric nerve). Peripheral nerves cannot be seen via ultrasound but are visible with an MRI; although MRI is multiplanar, it is more expensive, requires between 20 and 30 min to be completed, and does not allow a real-time evaluation. During fusion imaging, the MRI image reconstruction follows all ultrasound probe movements (real-time evaluation), making it possible to obtain oblique sections with a clear visualization of nerves [25].

## 4. Anatomical Considerations and Surgical Implications

Most endometriosis lesions of the USL involve the cervical section, which is the thickest (5–20 mm) part of the ligament [1]: We re-analyzed cases reported in Scioscia et al. [10] (USL involvement n = 1608) and found that 96.3% of endometriosis lesions occurred in the cervical section of the USL, with a slightly higher prevalence of lesions on the left USL (53.9% vs. 46.1%). In cases of cervical cancer spread, all the metastatic lesions of the USL obligately involve the proximal (cervical) part because they are a direct invasion of the tumor. Macroscopically, the cervical section of the USL is composed of dense connective tissue containing small blood vessels and small branches of the IHP [1]. These diseases that involve the USL may spread to nearby tissues like parametrium and paracervix in about 35% of cases [26].

**Figure 7:**
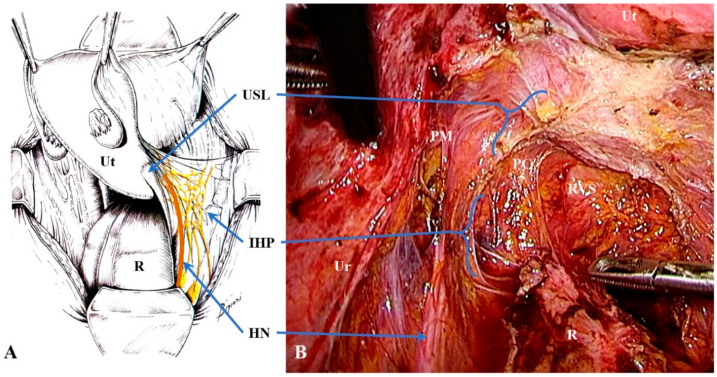
The hypogastric nerve (orange) and inferior hypogastric plexus (yellow) in an anatomical table (**A**); the image was modified from “Innervazione viscerale e somatica della pelvi femminile” by Ceccaroni, M, Fanfani F, Ercoli A, Scambia G; Ed. 2017, CIC Edizioni Internazionali) and surgery (**B**). In figure (**B**), the edge of the left uterosacral ligament that is close to the vagina and cervix is exposed after resection for endometriosis. Abbreviations: HN, hypogastric nerve; IHP, inferior hypogastric plexus; PC, paracervix; PM, parametrium; R, rectum; RVS, rectovaginal septum; Ur, ureter; USL, uterosacral ligament; Ut, uterus.

Parametrial and paracervical involvement makes surgery more complex and requires a specific surgical-anatomical preparation [9,15]. In fact, the paracervix and USL contain the pelvic splanchnic nerves, the distal part of the hypogastric nerve, and the IHP (Figure 7). The IHP is composed of sympathetic fibers from the hypogastric nerve and parasympathetic fibers from the splanchnic nerves and nerve anastomoses (which arise from the second-to-fourth sacral roots) [27]. Efferent fibers from the IHP travel to the rectum, uterus, rectovaginal ligament, and deep vesicouterine ligament. These efferent fibers are essential for bladder and rectal functionality so tailored nerve-sparing surgery became a standard approach for treating deep infiltrating endometriosis and cervical cancer [15,28,29,30].

**Figure 8:**
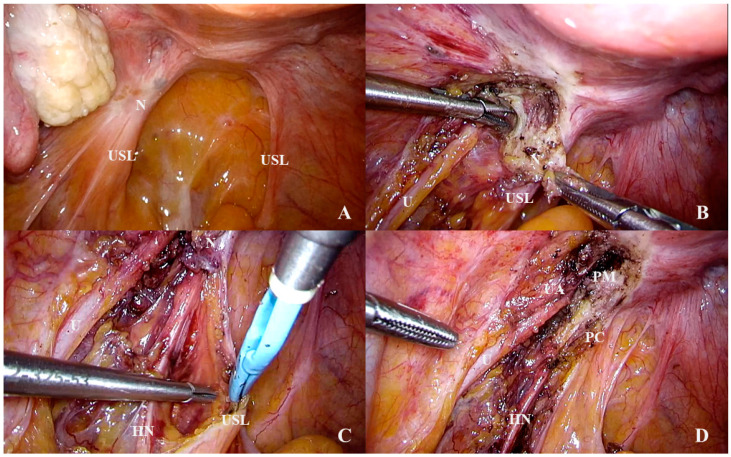
An isolated lesion of endometriosis of the left uterosacral ligament (**A**). During the dissection, the nodule was shown to extend laterally through the parametrium to the ureter (**B**) and inferiorly to the hypogastric nerve (**C**). After the complete removal of the nodule, the parametrium and the upper part of the paracervix was exposed (**D**). Abbreviations: HN, hypogastric nerve; N, endometriosis nodulus; PC, paracervix; PM, parametrium; U, ureter; UA, uterine artery; USL, uterosacral ligament.

Diseases of the USL, such as endometriosis and cervical cancer, may also involve nearby structures such as the ureter, the hypogastric nerve, and the IHP (Figure 8). These diseases, therefore, require expert surgery to avoid major complications, such as ureter fistulas, voiding dysfunction, or constipation.

From a surgical point of view, parametrium and paracervix, which is the upper part of the paracolpium (see above), contain structures that require different surgical approaches [31]. Namely, the parametrium contains the ureter and vessels that are usually isolated in advanced surgery (radical hysterectomy and deep infiltrating endometriosis) while the paracervix contains vessels (usually sacrificed whenever required) and nerves that should be, if possible, preserved in modern nerve-sparing radical surgery. However, unilateral resection of the HN, when the disease involves deeply the nerve and perineural spaces and neurolysis is not possible, is considered acceptable in the context of nerve-sparing surgery.

## 5. Discussion

Ultrasound is an accurate method for identifying the extent of pelvic endometriosis and cervical cancer. A correct visualization of the USL may improve the detection of lesions and their possible extension to the parametrium and the paracervix. Evaluating paracervical tissue is a key component of pre-operative assessment as it contains important structures, such as ureters and nerves, which may make surgery more complex. A multidisciplinary surgical approach may sometimes be required to avoid the long-term complications that can result from inadvertent damage to these structures.

The role of ultrasound in assessing USLs, parametrium, and paracervix has been discussed in the literature but in all cases the visualization of the USL was suggested to be obtained by pushing the probe to make the ligament closer to the transducer to improve the imagine quality [19,20,32]. In this article, we suggest placing the probe in the posterior vaginal fornix and then rotate of about 45° without pushing the transducer to also visualize structures that lie between the USL and the vagina (paracervix; proximal to the probe) and laterally to the USL (parametrium; distal to the probe). As we reported above, strongly pushing the probe to make the USL closer to the vagina may result in a displacement of the ligament, which can mask the actual extent of the lesion, perhaps to relevant structures like nerves and ureter. We reported some surgical and anatomical considerations that make clear the relevance of this anatomical area and the need for a correct visualization and diagnosis.

Another important consideration for ultrasound visualization is the experience level of sonographers, as demonstrated by high variability in the detection of USL endometriosis (sensitivity ranges from 0.10 to 0.89) [33]. According to recent studies on the learning curve for using transvaginal ultrasound in cases of deep infiltrating endometriosis [34,35], the USL appears to be the most difficult structure to explore accurately: lower sensitivity/specificity values are reported for the USL than for other structures, and clinicians need to perform a higher number of scans to achieve proficiency. A systematic approach, such as the one proposed by the International Deep Endometriosis Analysis (IDEA) group [5], may improve the diagnostic accuracy and predictive value of ultrasound [36], and we therefore look forward to the findings of the ongoing multicenter IDEA study.

## 6. Conclusions

This article highlights the importance of a correct visualization by ultrasound not only of the USL but the contemporary assessment of the paracervix and parametrium. This can be achieved during transvaginal sonographic dynamic real-time assessment of the USL without pushing the probe to assess the depth of the lesion that may extend to structures like nerves. Paracervical and parametrial infiltration represents an important aspect in preoperative assessment to optimize preoperative counselling and informed consent (for risk of postoperative bladder dysfunctions), and to involve expert surgeons during the operation. In this paper, we attempted to establish a common terminology between imaging diagnostic and modern surgical anatomy to overcome discrepancies in clinical and research settings.

## Supplementary Materials

The following are available online at https://www.mdpi.com/2077-0383/10/3/437/s1, Video S1. Ultrasound visualization during surgery of the uterosacral ligament, paracervix, and parametrium. The hyperechoic line (arrow) is the suture that was placed on the uterosacral ligament. Abbreviations: C, cervix; PC, paracervix; PM, parametrium; USL, uterosacral ligament; V, vagina wall. Video S2. Displacement of the uterosacral ligament during the ultrasound when the probe is strongly pushed. Video S3. Visualization of the small vessels within the lowest part of the uterosacral ligament using color Doppler.
