# Supplementary material for: Ultrasound of the Uterosacral Ligament, Parametrium, and Paracervix: Disagreement in Terminology between Imaging Anatomy and Modern Gynecologic Surgery
Source: J Clin Med. 2021 Jan 23;10(3):437. doi: 10.3390/jcm10030437 (PMC7865545; doi:10.3390/jcm10030437)
Supplement: Supplementary file 1 [file jcm-10-00437-s001.zip › jcm-1037559-supplementary/Video legends.pdf]

Video S1. Ultrasound visualization during surgery of the uterosacral ligament, paracervix, and parametrium. The hyperechoic line (arrow) is the suture that was placed on the uterosacral ligament.

Abbreviations: C, cervix; PC, paracervix; PM, parametrium; USL, uterosacral ligament; V, vagina wall.

Video S2. Displacement of the uterosacral ligament during the ultrasound when the probe is strongly pushed.

Video S3. Visualization of the small vessels within the lowest part of the uterosacral ligament using color Doppler.
